# Anesthetic management of tracheal laceration from traumatic dislocation of the first rib: a case report and literature of the review

**DOI:** 10.1186/s12871-019-0812-9

**Published:** 2019-08-09

**Authors:** Penghui Wei, Dong Yan, Jiapeng Huang, Lili Dong, Ying Zhao, Fei Rong, Jing Li, Wenxi Tang, Jianjun Li

**Affiliations:** 1 Department of Anesthesiology, Qilu Hospital of Shandong University (Qingdao), No.758 Hefei Road, Qingdao, People’s Republic of China; 2 Department of Anesthesiology & Perioperative Medicine, University of Louisville, Louisville, KY USA

**Keywords:** Tracheobronchial lacerations, First rib, Anesthetic management, Flexible bronchoscopy

## Abstract

**Background:**

Tracheobronchial lacerations from trauma can be life-threatening and present significant challenges for safe anesthetic management. Early recognition of tracheal injuries and prompt airway control can be lifesaving.

**Case presentation:**

A 56-year-old man with no significant medical history presented with difficulty breathing after a blunt trauma to his chest to the emergency room and was diagnosed with dislocation of the first rib and tracheal laceration after a chest tomography (CT) study. Subcutaneous emphysema in neck area quickly worsened indicating continuous air leak. Emergent surgical repair was scheduled. General anesthesia with maintaining spontaneous ventilation was performed and a 5.5 mm endotracheal tube was placed under the guidance of flexible bronchoscopy. Depth of anesthesia was maintained to achieve a Bispectral Index Score of 40–60. Once the offending first rib was removed, a 7.5 mm endotracheal tube was inserted distal to the laceration site with the guidance of flexible bronchoscopy. Once confirmed location of the endotracheal tube, cisatracurium was administered intravenously and the patient was managed on mechanical ventilation with interval positive pressure ventilation. The operation was successful and he was transferred to the ICU intubated. He then received elective surgical repairs for sternum fracture, multiple rib fractures and hemopneumothorax under general anesthesia on day 5 after the first surgery and was extubated on postoperative day 7. The subsequent course was uneventful. Comprehensive rehabilitation was done for 2 weeks and he was discharged home on postoperative day 41.

**Conclusions:**

Early diagnosis and multidisciplinary collaborations are keys to the successful management of this patient. Flexible bronchoscopy is particularly useful in airway management for urgent trachea tracheal laceration repair.

## Background

Traumatic tracheobronchial lacerations are relatively uncommon with incidence between 0.5 and 2% among patients with multiple injuries [1]. About 19% of tracheobronchial lacerations occur in the trachea only, 32% are in the left main stem bronchus only, and 47% are in the right main stem bronchus only [2]. Tracheobronchial lacerations are the second most common causes of death and more than 75% of patients die before they arrive to the emergency department [3, 4]. Tracheobronchial lacerations present unique challenges to the anesthesiologist, early recognition and prompt airway control are key for survival.

Tracheobronchial lacerations could be caused by blunt trauma, penetrating trauma or iatrogenic injuries from emergency intubations, multiple intubation attempts, and over-inflation of the tracheal cuff [1, 5]. Acute traumatic tracheal injury is rare to be seen by anesthesiologists because tracheobronchial lacerations usually result in acute airway obstruction and death at the scene of an accident or crime [6]. In recent years, more patients with tracheobronchial lacerations presented to the emergency department due to better pre-hospital evacuation procedures and heightened trainings [4]. Tracheobronchial lacerations from traumatic dislocation of the first rib is exceedingly rare.

The anatomical location of the first rib determines that significant dislocation could result in injuries of subclavian vessels, trunks of the brachial plexus, cervicothoracic ganglion, and trachea [7]. The first rib is not commonly dislocated in trauma because its articulations at the T1 vertebra and the manubrium are stabilized by strong ligaments [8]. In rare cases, traumatic first rib dislocations could injure subclavian artery, cervicothoracic ganglion, brachial plexus and trachea [7].

## Case presentation

**Fig. 1:**
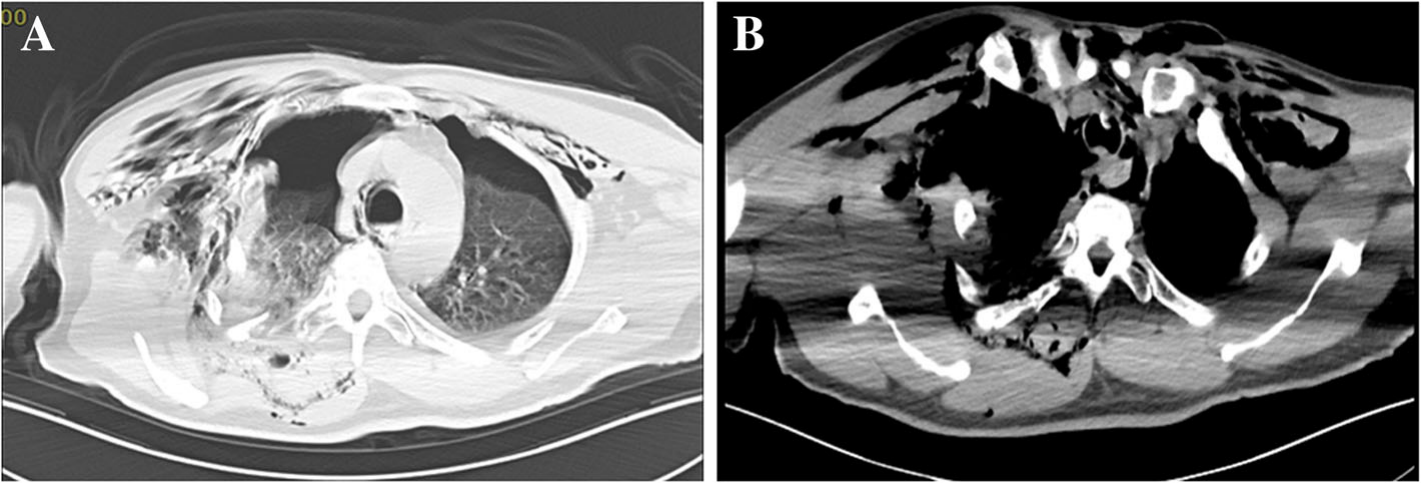
Thoracic CT scan showing massive subcutaneous emphysema, pneumomediastinum, multiple rib fractures, bilateral hemopneumothorax and compressive pneumothorax on pulmonary (**a**) and mediastinal (**b**) window

**Fig. 2:**
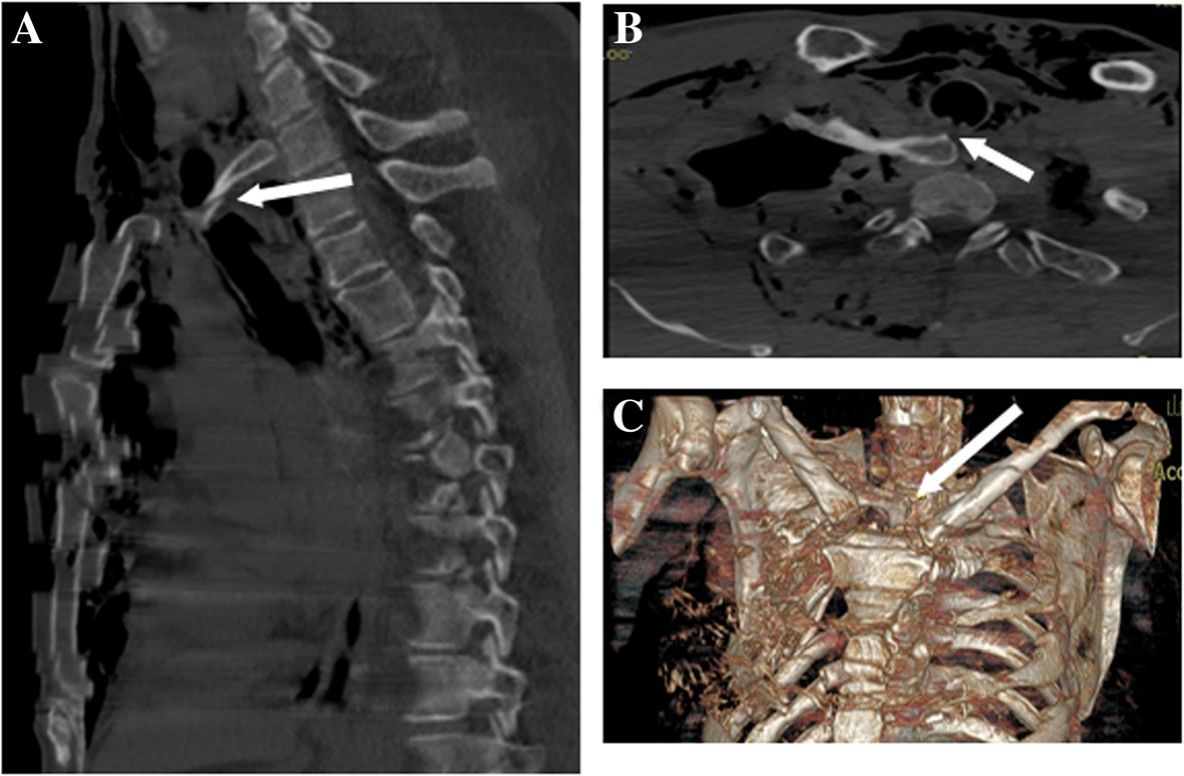
CT scans of multi-slice technique showing the tracheal laceration secondary to the dislocation of right first rib. **a** Sagittal CT image of the chest. **b** Axial CT image of the chest. (C) Volume rendering of thorax

**Fig. 3:**
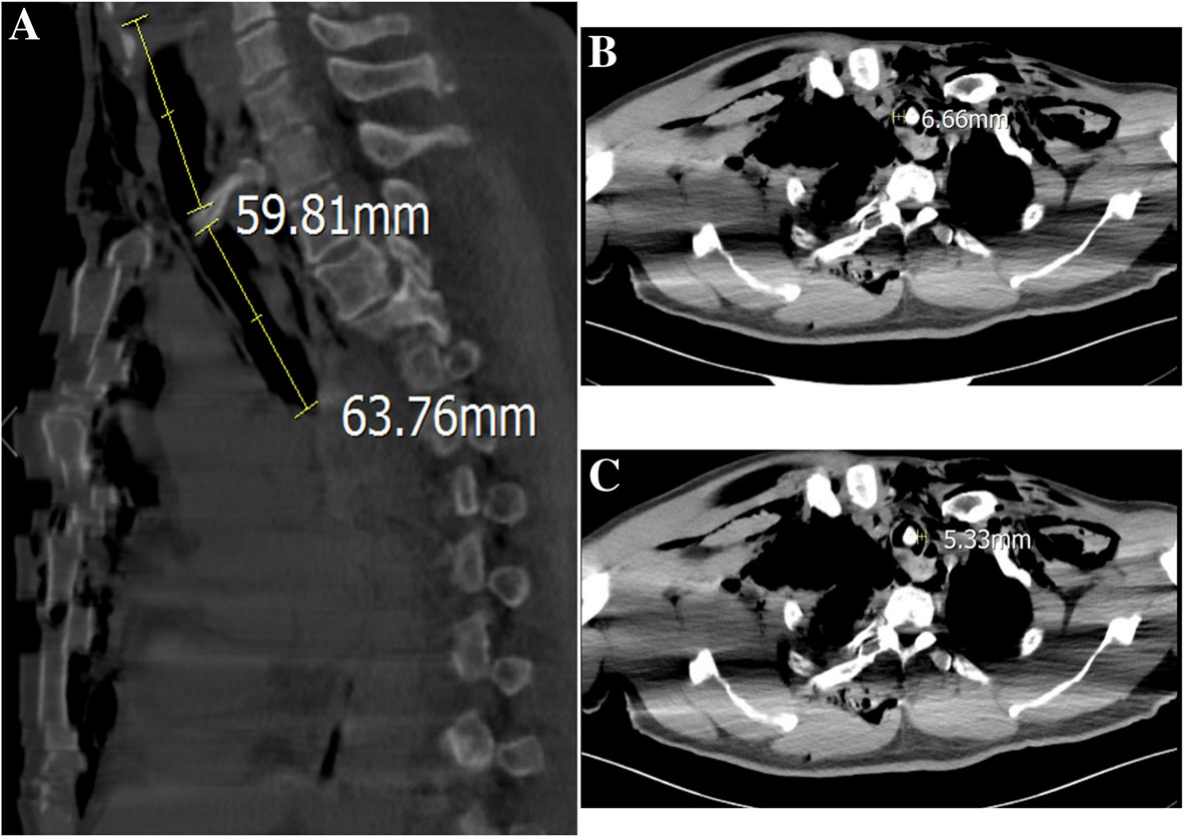
Preoperative evaluation of Tracheobronchial lacerations by a high-resolution CT. **a** Sagittal CT image of the chest showing the posterior tracheal wall laceration up to 59.81 mm below the glottis and 63.76 mm above the carina. **b**, **c** Axial CT image of the chest showing the bone shadow in the trachea; the residual largest cavity of the trachea on the left was 5.33 mm in diameter and 6.66 mm on the right

**Fig. 4:**
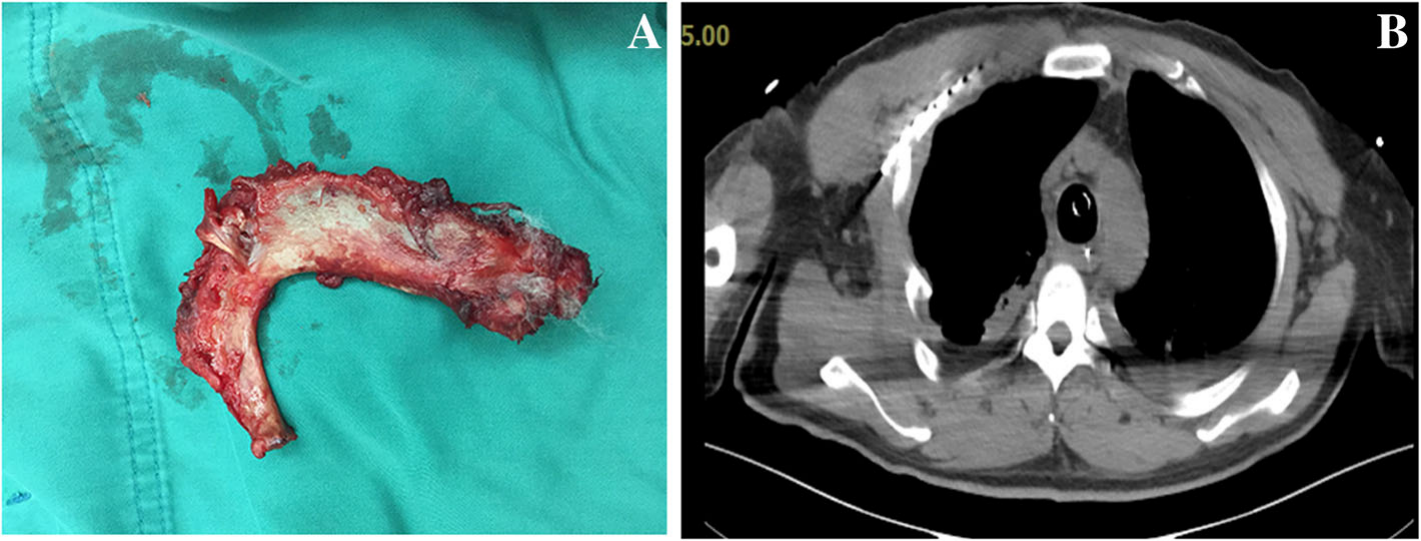
**a** The right first rib was removed. **b** Postoperative chest CT-scan showing the integrity of posterior tracheal wall

Written informed consent for the publication of this case was obtained from the patient. A 56-year-old man (height, 165 cm; weight, 70 kg) with no significant medical history presented to the emergency room complaining of right chest pain, chest congestion and shortness of breath after sustaining blunt trauma to the right chest. His vital signs were as follows: heart rate (HR), 91 beats/min; respiratory rate (RR), 34 breaths/min; blood pressure (BP), 108/71 mmHg; and initial pulse oximetry saturation (SpO_2)_ of 85%. Arterial blood gas: PH 7.35, PaCO_2_ 47 mmHg, and PaO_2_ 49 mmHg. Flail chest and paradoxical breathing were evident. Significant subcutaneous emphysema in neck and anterior chest area was diagnosed with marked crepitus throughout. Computed Tomography (CT) scan showed massive subcutaneous emphysema, pneumomediastinum, multiple rib fractures, bilateral hemopneumothorax and compressive pneumothorax (Fig. 1 a and b). The sternocostal articulation displacement was seen and the sternal portion of the right first rib penetrated the posterior tracheal wall above the carina (Fig. 2). Chest tube was inserted to decompress pneumothoraxes and hemopneumothorax, and breathing difficulties were alleviated. Unfortunately, rapid worsening of subcutaneous emphysema indicated continuous air leak from laceration after 3 h. The patient developed respiratory distress and became hemodynamically unstable. Emergent CT demonstrated that the right first rib penetrated the posterior tracheal wall up to approximately 6 cm below the glottis and 6 cm above the carina (Fig. 3 a). The first rib divided the trachea into two parts, 5.3 mm in diameter on the left and 6.6 mm on the right (Fig. 3 b and c). The patient was quickly transferred to the operation room. He was agitated, in respiratory distress and his vital signs were: HR 108 beats/min; RR 30 breaths/min; BP 90/58 mmHg and SpO_2_ 80%. We maintained the hemodynamic stability with intravenous phenylephrine. A fiberoptic bronchoscope was immediately available with different sized endotracheal tubes. General anesthesia was induced with midazolam 2 mg, fentanyl 0.05 mg and 2%~ 5% sevoflurane while maintaining spontaneous ventilation. A sterile flexible fiberoptic bronchoscope loaded with a 5.5 mm endotracheal tube (outer diameter 7.3 mm) identified the laceration of the trachea and the endotracheal tube was advanced distally past the laceration site. He was spontaneously breathing with fraction of inspired oxygen 100%, tidal volume 330 ml, frequency 30, SpO_2_ 95% and end-tidal carbon dioxide partial pressure (PetCO_2_) 40 mmHg. Depth of anesthesia was maintained to achieve a Bispectral Index Score of 40–60. The thoracic cavity was opened to expose the right first rib by the surgical team and the right first rib was removed approximately 30 min later (Fig. 4 a). A 7.5 mm endotracheal tube was then exchanged and positioned distally to the laceration with the guidance of flexible bronchoscopy. Once confirmed location of the endotracheal tube, cisatracurium 14 mg and fentanyl 0.15 mg were administered intravenously. The patient was managed on mechanical ventilation with interval positive pressure ventilation. The respiratory parameters were: fraction of inspired oxygen 60%, tidal volume 550 ml, frequency12, airway peak pressure 22 cm H_2_O, SpO_2_ 98% and PetCO_2_ 38 mmHg. Direct surgical repair of the tracheal laceration was successful and he was transferred to the ICU intubated. In ICU, he was managed on the ventilator with synchronized intermittent mandatory ventilation and continuous positive airway pressure. In order to improve pulmonary function, elective surgical repairs of sternum fracture, multiple rib fractures and hemopneumothorax under general anesthesia were performed on day 5 after first surgery and the patient was extubated on postoperative day 7. Repeated CT demonstrated the integrity of tracheal wall (Fig. 4 b). Comprehensive rehabilitation was done for 2 weeks and he was discharged home on postoperative day 41.

## Discussion and conclusions

In blunt trauma, approximately 80% of tracheobronchial lacerations occur near the carina at the distal trachea, typically in the posterior trachea wall due to lack of cartilaginous support [6]. Tracheal wall laceration from the first rib is a rare complication of blunt chest trauma. Early diagnosis and treatment are associated with fewer morbidity and less complications including infection and tracheobronchial stricture [9].

The diagnosis of tracheobronchial lacerations is based on high clinical suspicion and signs of subcutaneous emphysema, pneumothorax, or pneumomediastinum [10, 11]. CT scan of the chest should be the initial screening tool in hemodynamically stable patients suffering from multiple chest trauma [12]. A persistent pneumothorax with large air leak from a well-placed chest tube should raise the suspicion of potential airway injury. Other important radiographic findings that are associated with tracheobronchial tears include incorrect location or overdistention of the endotracheal tube cuff [13]. The gold standard for diagnosis is bronchoscopy, which can identify the details of the laceration and guide the accurate positioning of the endotracheal tube [11]. However, tracheobronchial tears may not be visible if the tracheal mucosa remains intact or is sealed by fibrin. In addition, expertise and availability of bronchoscopy can further delay the diagnosis [14]. On a supine CT examination, a classic “fallen-lung sign” is reported as being specific for a bronchial tear, which refers to the peripheral, rather than central, lung collapse occurring when the normal central bronchial anchoring attachments of the lung are disrupted. The collapsed lung falls toward the dependent portion of the hemithorax and is thus seen posteriorly when the patient is supine as during CT examination [13, 14]. No fallen-lung sign was observed in our case because the laceration site was located in the trachea not the mainstem bronchus.

**Fig. 5:**
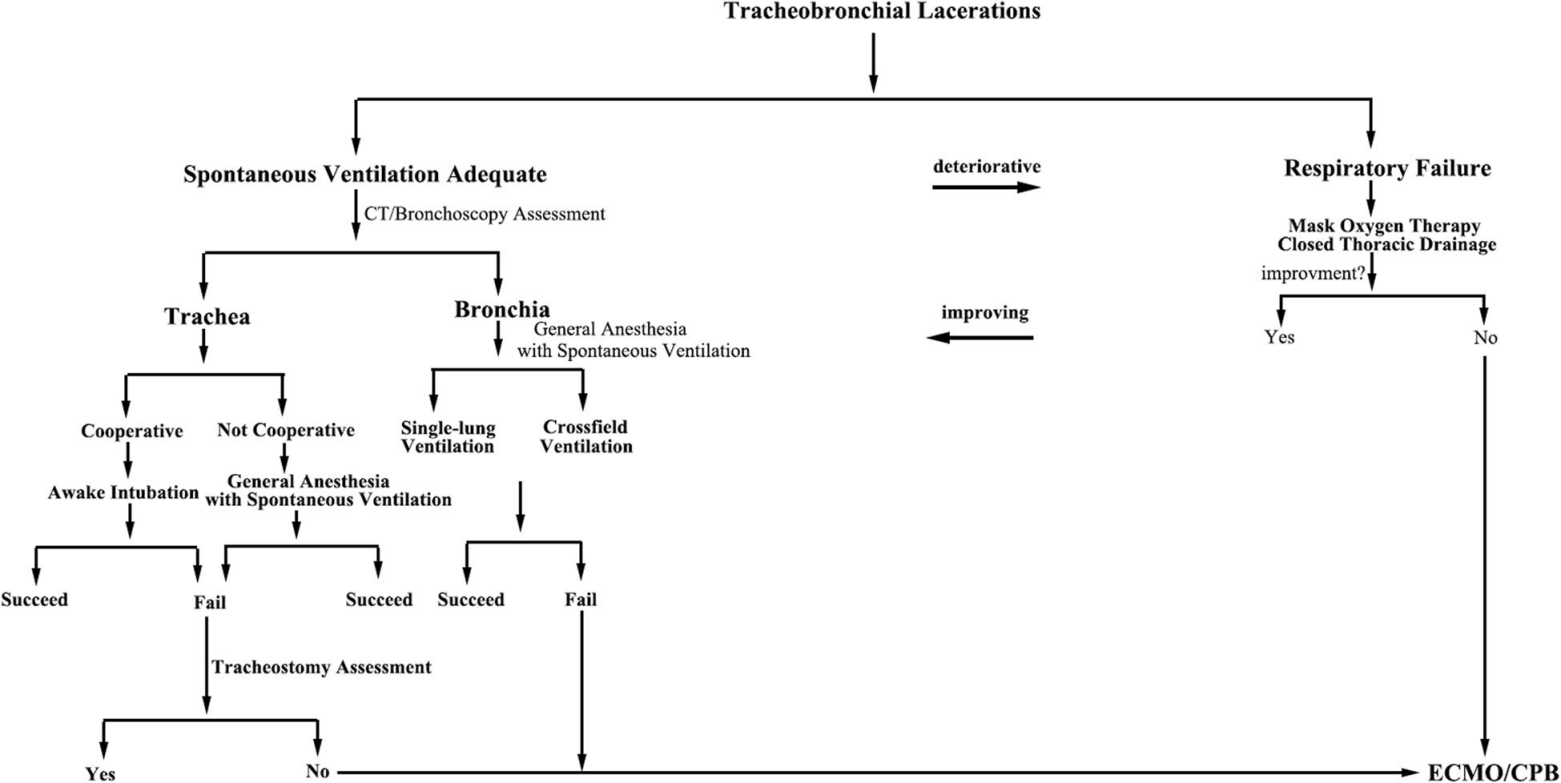
The proposed difficult airway algorithm in patients with tracheobronchial lacerations

Management of small lacerations (lesions smaller than 1 cm) without surgical intervention may be possible if the endotracheal tube is able to stent the laceration and allow for wound healing [5, 15]. Lacerations larger than 1 or 2 cm or with extensive pneumomediastinum, progressive subcutaneous emphysema, and deteriorative ventilation should be addressed with primary repair [10]. In our case, we performed surgical removal of the first rib and repair of the trachea because the dislocated first rib could cause further tearing of the trachea and the chance of spontaneous healing was not likely. Patients with high suspicion of a tracheal laceration or clinically unstable with rapid drop in oxygenation, should be intubated immediately, under spontaneous ventilation with the guidance of a flexible bronchoscopy [1]. Other ventilatory management options include awake intubation with local anesthetic infiltration, cricothyrotomy, tracheostomy, extracorporeal membrane oxygenation (ECMO), cardiopulmonary bypass (CPB) and cross-field ventilation [4, 16]. In patients with tracheobronchial lacerations, we propose the following difficult airway algorithm (Fig. 5). Awake intubation with local anesthetic infiltration may be a safer option in many difficult airway management [4]. However, the patient was agitated and cannot cooperate when he was transferred to the operation room. In order to prevent larger tears caused by unintended movements during intubation, general anesthesia with spontaneous ventilation was performed. Our patient was not a candidate for either cricothyrotomy or tracheostomy due to the presence of the first rib and the position of the laceration. Crossfield ventilation and single-lung ventilation are generally preferred for patients with the carinal or bronchial injuries [14, 17]. Our patient was intubated with a small single-lumen tube initially with bronchoscopy guidance and ECMO was available in the operating room as backup.

Veno-venous ECMO has proven to be an effective therapy in patients with tracheobronchial lacerations and is difficult to intubate and ventilate [18]. ECMO allows surgical repair by providing adequate oxygenation while minimizing the risk of positive pressure ventilation on tracheobronchial leakage and mechanical dehiscence [19]. The use of ECMO instead of traditional cardiopulmonary bypass technique in tracheal surgery was supported by the lack of intracranial hemorrhage and unstoppable bleeding associated with high-dosed anticoagulants [16, 18].

Successful management of Tracheobronchial lacerations requires constant communications between the anesthesiologist and the surgeon. A multidisciplinary approach and effective communication led to successful outcome in the case.

In summary, we presented a rare case of tracheal laceration from a dislocated first rib. We highlighted the importance of multi-dimensional analysis of a high-resolution CT in diagnosing the tracheal laceration caused by the first rib. Flexible bronchoscopy is particularly useful for successful difficult airway management in urgent tracheobronchial laceration. The importance of effective communication between anesthesiologists and surgeons can’t be over-emphasized in these challenging cases.

## Data Availability

All data related to this case report are contained within the manuscript.

## Abbreviations

BP: Blood pressure
CPB: Cardiopulmonary bypass
CT: Computed tomography
ECMO: Extracorporeal membrane oxygenation
HR: Heart rate
_ICU_: Intensive care unit
ID: Internal diameter
PetCO2: end-tidal carbon dioxide partial pressure
RR: respiratory rate
SpO_2_: Pulse oximetry saturation
